# Alterations of Regional Homogeneity in the Mild and Moderate Stages of Parkinson’s Disease

**DOI:** 10.3389/fnagi.2021.676899

**Published:** 2021-07-21

**Authors:** Junli Li, Haiyan Liao, Tianyu Wang, Yuheng Zi, Lin Zhang, Min Wang, Zhenni Mao, ChenDie Song, Fan Zhou, Qin Shen, Sainan Cai, Changlian Tan

**Affiliations:** Department of Radiology, The Second Xiangya Hospital, Central South University, Changsha, China

**Keywords:** Parkinson’s disease, resting-state functional MRI, regional homogeneity (ReHo), Hoehn and Yahr stage, early diagnosis

## Abstract

**Objectives**: This study aimed to investigate alterations in regional homogeneity (ReHo) in early Parkinson’s disease (PD) at different Hoehn and Yahr (HY) stages and to demonstrate the relationships between altered brain regions and clinical scale scores.

**Methods**: We recruited 75 PD patients, including 43 with mild PD (PD-mild; HY stage: 1.0–1.5) and 32 with moderate PD (PD-moderate; HY stage: 2.0–2.5). We also recruited 37 age- and sex-matched healthy subjects as healthy controls (HC). All subjects underwent neuropsychological assessments and a 3.0 Tesla magnetic resonance scanning. Regional homogeneity of blood oxygen level-dependent (BOLD) signals was used to characterize regional cerebral function. Correlative relationships between mean ReHo values and clinical data were then explored.

**Results**: Compared to the HC group, the PD-mild group exhibited increased ReHo values in the right cerebellum, while the PD-moderate group exhibited increased ReHo values in the bilateral cerebellum, and decreased ReHo values in the right superior temporal gyrus, the right Rolandic operculum, the right postcentral gyrus, and the right precentral gyrus. Reho value of right Pre/Postcentral was negatively correlated with HY stage. Compared to the PD-moderate group, the PD-mild group showed reduced ReHo values in the right superior orbital gyrus and the right rectus, in which the ReHo value was negatively correlated with cognition.

**Conclusion**: The right superior orbital gyrus and right rectus may serve as a differential indicator for mild and moderate PD. Subjects with moderate PD had a greater scope for ReHo alterations in the cortex and compensation in the cerebellum than those with mild PD. PD at HY stages of 2.0–2.5 may already be classified as Braak stages 5 and 6 in terms of pathology. Our study revealed the different patterns of brain function in a resting state in PD at different HY stages and may help to elucidate the neural function and early diagnosis of patients with PD.

## Introduction

Parkinson’s disease (PD) was first described by James Parkinson in 1817 (Hurwitz, 2017) and is now the second most common neurodegenerative disease after Alzheimer’s disease (Khan et al., 2019). The worldwide prevalence of PD is approximately 0.3% in the general population above 40 years of age (Pringsheim et al., 2014). It is estimated that the number of people suffering from PD in China will rise from 1.99 million in 2005 to 5 million in 2030, accounting for almost half of the total global population of PD patients (Li G. et al., 2019). PD is a multi-system disorder that is manifested by a range of motor symptoms, including rest tremor, stiffness, bradykinesia, and postural instability, as well as concomitant non-motor symptoms, such as hyposmia, depression, anxiety, cognitive dysfunction, and sleep disorders (Shrestha et al., 2017; Reich and Savitt, 2019; Singh et al., 2020a; Zahra et al., 2020). With an aging population, the prevalence of PD will undoubtedly reduce the quality of life for the elderly and create a significant medical burden on human society.

The core pathology of PD is considered to involve the deposition of Lewy bodies and the destruction of dopamine neurons in the substantia nigra pars compacta of the midbrain, thus leading to disruption of the basal ganglia and the initiation of motor symptoms (Rai et al., 2016, 2017, 2019; Singh et al., 2020b). In Braak’s staging system, the pathology of PD can be divided into six stages according to the presence of Lewy bodies; the deposition of Lewy bodies begins in the dorsal IX/X motor nucleus or intermediate reticular zone and reaches the lower brain stem nuclei and eventually extends upwards to the neocortex (Kon et al., 2020). However, when motor symptoms appear, the loss of dopaminergic neurons in the substantia nigra has already reached at least 60% (Hornykiewicz, 2006), thus corresponding to Braak stage 3 or 4. At Braak stages 1 or 2, patients with PD often have only motor symptoms with no typical characteristics on conventional imaging. Consequently, these patients tend to be diagnosed with other neurological diseases, such as depression, anxiety disorders, Alzheimer’s disease, and sleep disorders. Hence, identifying PD patients at an early stage is critical for the clinical management and treatment of this disease.

Resting-state functional magnetic resonance imaging (rs-fMRI) can measure continuous cerebral activity by recording blood oxygen level-dependent (BOLD) signals and is one of the main major imaging methods used to study the neurobiological mechanisms of PD. rs-fMRI can be divided into functional separation and functional integration. Functional separation predominantly investigates the characteristics of regional neural spontaneous activity, such as the amplitude of low frequency fluctuation analysis (ALFF) and regional homogeneity analysis (ReHo). In contrast, functional integration emphasizes the correlations and interactions between remote brain regions by functional connectivity (FC) or network analysis, such as independent component analysis (ICA), FC density analysis (FCD), seed-based FC analysis, and graph analysis (Zuo and Xing, 2014; Lv et al., 2018).

Functional integration is the primary method used to explore the activity of the human brain. However, functional separation can potentially influence the global network dynamics. For example, changes in the ReHo value are thought to cause alterations of remote FC (Jiang and Zuo, 2016). ReHo values are determined by the Kendall coefficient of concordance (KCC) in between the BOLD time-series, and describes the homogeneity of a given voxel and the most adjacent 26 voxels (Yang et al., 2020). ReHo values can be regarded as indicators of network centrality to represent the significance of nodes in functional connectomes within the cerebrum (Jiang and Zuo, 2016; Lv et al., 2018).

A multitude of researchers has attempted to use magnetic resonance to study the early phases of PD. For example, Claassen et al. (2016) identified asymmetric cortical atrophy in the left cerebrum, particularly in the left insula and olfactory sulcus. In a series of rs-fMRI studies, a number of cerebral areas were proposed to be related to early PD (Long et al., 2012; Yang et al., 2013; Fioravanti et al., 2015; Xu et al., 2019). These studies made a significant contribution to the possible cerebral structural or functional changes in early PD. Nevertheless, these results were inconsistent. We hypothesize that this inconsistency is because PD patients at different stages correspond to different cerebral alteration patterns.

Based upon the Hoehn and Yahr (HY) scale, created in 1967, the “modified HY scale” features 0.5 increments and has been widely used to evaluate the clinical progression of PD (Hoehn and Yahr, 1967; Goetz et al., 2004). Guan et al. (2019) coupled various oscillation frequencies in PD and observed progressive oscillation-specific nodal alterations from the early to middle stages of PD. Further research based on the ALFF and FC of PD patients with different HY stages indicated a higher function default mode network(DMN) in stage II (Luo et al., 2015). More recent research has focused on the use of structural MRI to investigate PD patients at different HY stages. Compared to a mild PD group, a group of patients with moderate PD showed an increased cortical thickness in a number of brain areas, including the temporal pole, isthmus cingulate cortex, superior frontal cortex, fusiform gyrus, insula lobe, and the inferior temporal cortex (Gao et al., 2018). Therefore, we hypothesized that ReHo values will vary as PD progresses. In this study, we used ReHo analysis to compare changes in cerebral function at various HY stages of Parkinson’s disease (PD). We also investigated how the pathogenesis of PD changed with different stages.

## Materials and Methods

### Subjects

All PD patients and healthy subjects were recruited between December 2015 and October 2020. This research was authorized by the Ethics Committee of the 2nd Xiangya Hospital. All patients were diagnosed by two neurologists according to the Movement Disorder Society (MDS) PD criteria (Postuma et al., 2015). For both PD patients and normal controls, we obtained a range of demographic and clinical information, including age, gender, education, the 17-item Hamilton Depression Scale (HAMD-17) score, and the Mini-Mental State Exam (MMSE) score. For PD patients, we recorded disease duration, the Unified Parkinson’s Disease Rating Scale score (UPDRS, featuring a motor component named UPDRS-III), and the HY Scale score. Patients who met the following criteria were included: (1) patients satisfied the MDS PD criteria for clinically established PD; (2) patients were right-handed; (3) patients had stopped taking anti-PD drugs for 12 h; and (4) patients had motor signs and symptoms at an HY stage of 1.0–2.5. Subjects were excluded if they: (1) had other diseases that could potentially affect brain function, such as atypical Parkinsonism, depression, cerebral trauma, stroke, and other diseases of the neurological system, *n* = 3; (2) had contraindications to MRI or were unable to cooperate with an MRI scan and clinical scales, *n* = 6; or (3) had an MMSE score less than the corresponding education degree, *n* = 3. MMSE scores of >17 for illiterate subjects, >20 for 1–6 years of education, and >23 for 7 or more years of education, were defined as normal MMSE scores (Li et al., 2016); (4) had excessive head motion (greater than 0.5 mm in transformation and 0.5 degrees in rotation), *n* = 4; and (5) had not withdrawn from anti-Parkinson drugs, *n* = 6. In total, 75 PD patients (with HY stages of 1.0–2.5) were included in this research. PD patients with an HY stage of 1.0–1.5, corresponding to unilateral motor symptoms, were defined as having mild PD (PD-mild, *n* = 43). Patients with an HY stage of 2.0–2.5, corresponding to bilateral motor symptoms, were defined as having moderate PD (PD-moderate, *n* = 32). Thirty-seven right-handed healthy subjects that were matched for age, sex, and education, were recruited as healthy controls (HC, *n* = 37).

### Image Acquisition

Imaging data were acquired by a Siemens 3.0T MRI scanner by a radiologist at the Radiology Department of the 2nd Xiangya Hospital, Central South University. During MRI scanning, each individual was asked to lie in a supine position wearing earmuffs to reduce the sound of the MRI system. The patients also had foam pads around their heads to minimize head movement. All subjects were then informed to remain relaxed during rs-fMRI acquisition, with their eyes closed but avoiding sleep and active thought. Rs-fMRI images were acquired by an Echo Planar Imaging (EPI) sequence with the following parameters: echo time (TE) = 25 ms; repetition time (TR) = 2,500 ms; voxel size = 3.75 × 3.75 × 3.5 mm; flip angle (FA) = 90°; field of view (FOV) = 240 × 240 mm^2^, data matrix = 64 × 64; slice gap = 0 mm; slice thickness = 3.5 mm; 39 interleaved slices and 200 volumes. T1WI three-dimensional magnetization- prepared rapid acquisition gradient echo (T1WI-3D-MP RAGE) images were acquired with the following parameters: TE = 2.01 ms; TR = 1900 ms; voxel size = 1 × 1 × 1 mm; slice thickness = 1 mm; FA = 9°; FOV = 256 mm × 256 mm; 176 continuous sagittal slices.

### MRI Data Pre-processing

The rs-fMRI data were preprocessed by the Resting State fMRI Data Analysis Toolkit (RESTplus) software version 1.21 (Xi-Ze et al., 2019)^1^; this is a software package that is based on Statistical Parametric Mapping 8 (SPM8) on the MATLAB R2014b platform (The MathWorks Inc., Natick, MA, USA). Pre-processing involved seven steps, as follows: (1) converting data from digital imaging and communications in medicine(DICOM) to neuroimaging informatics technology initiative(NIFTI); (2) eliminating the initial 10 volumes; (3) slice timing; (4) realignment and the evaluation of head movement (exclusion criteria: >0.5 mm in transformation and >0.5 degrees of rotation); (5) spatial normalization (this was divided into three steps: setting the origin to anterior commissure for each patient’s T1WI-3D-MP RAGE; registration of high resolution T1WI to mean functional MRI, division of the T1WI with Diffeomorphic Anatomical Registration *via* the Exponentiated Lie Algebra (DARTEL; Ashburner, 2007) toolkit, the generation of a group template; transformation and normalization of the resulting aligned data to the Montreal Neurological Institute (MNI) space with the segmented gray matter from DARTEL); (6) removal of the linear trend generated from MRI or other factors; (7) nuisance covariate regression with six head motion parameters, white matter, and cerebrospinal fluid signal (Yan et al., 2013); and (8) filtering with a bandpass of 0.01−0.08 Hz.

### Regional Homogeneity

Next, we used RESTplus software to calculate a voxel-wise ReHo map for each patient. A z-transformation was then performed by deducting the mean value of the entire brain from the resulting ReHo map and dividing by the global standard deviation. In addition, we smoothened the ReHo map with a full width at a half maximum (FWHM) Gaussian kernel of 6 mm. The standardized ReHo Z-maps were then used for correlative analysis while the smoothened ReHo maps were used for statistical analysis to investigate regional homogeneity.

### Statistical Analysis for Demographic and Clinical Information

First, we tested data for normality with the Shapiro–Wilk Test; Levene’s Test was used to evaluate the homogeneity of variance. Patient age and the number of years of education were distributed normally and showed homogeneity of variance; the other clinical data did not comply with these stipulations (*p* < 0.05). Differences in age and education degree across the PD-mild, PD-moderate, and HC groups were compared by analysis of variance (ANOVA), while the independent *t*-test was used to identify differences between the entire PD group and the HC or PD groups. Due to the qualitative nature of the data, gender distribution among/between groups was tested by the Pearson Chi-squared test. Due to the non-normal distribution of data, differences in UPDRS, UPDRS-III, and disease duration, between the PD groups were compared with the Mann–Whitney Wilcoxon test. Differences in the MMSE and HAMD-17 scores across the three groups, and between the PD groups, were compared with the Kruskal–Wallis test and the Mann–Whitney Wilcoxon test, respectively. We also attempted to identify correlations among the clinical data. These analyses were conducted by IBM SPSS statistical analysis software (version 25.0; SPSS Inc. Chicago, IL, USA).

### Statistical Analysis for Regional Homogeneity and Correlative Analysis

One-way analysis of covariance (ANCOVA) was used to compare differences between the smoothened ReHo maps created for the PD-mild, PD-moderate, and HC groups, with age, gender, and education, serving as covariates. Significant differences were generated among the three groups (voxel-level *p* < 0.005; minimal cluster size >24 voxels; corresponding to *p* < 0.05 for a two-tail test as corrected by the AlphaSim program). In order to investigate the significant brain regions, we used a *post hoc* two-sample *t*-test to compare differences between each pair of the three groups (corrected by the AlphaSim program with a voxel-level *p* < 0.005; cluster-level *p* < 0.05 for a two-tail test and a cluster size >24 voxels). Brain regions that showed significant differences in the ANCOVA were extracted as masks so that we could investigate the correlative relationships between mean ReHo values and clinical data in the PD groups. Spearman’s correlation coefficient was calculated and the threshold of significance was set to *p* < 0.05 (corrected by Bonferroni’s correction). Correlation analysis was performed by SPSS version 25.0.

## Results

### Demographics and Clinical Characteristics

Table 1 summarizes the demographic information and clinical characteristics of the three groups. There were no significant differences between the three groups in terms of age, gender, years of education, and MMSE scores (*p* > 0.05). In our study, we excluded subjects with depression. However, we observed a significant difference in the HAMD-17 scores when compared between the PD and HC groups (*p* < 0.001); there was no significant difference when comparing between the PD-mild and PD-moderate groups (*p* = 0.060). The PD-moderate group had significantly higher UPDRS scores and a significantly longer disease duration than the PD-mild group (*p* = 0.001 and *p* = 0.012, respectively). Correlation analysis revealed a positive correlation between the following clinical parameters in the PD groups: MMSE scores with years of education (*r* = 0.621, *p* < 0.001); disease duration with UPDRS scores (*r* = 0.396, *p* < 0.001) and UPDRS-III scores (*r* = 0.382, *p* = 0.001); HAMD scores with UPDRS scores (*r* = 0.579, *p* < 0.001) and UPDRS-III scores (*r* = 0.444, *p* < 0.001); HY stages with disease duration (*r* = 0.323, *p* = 0.005), UPDRS scores (*r* = 0.576, *p* < 0.001), UPDRS-III scores (*r* = 0.609, *p* < 0.001), and HAMD scores (*r* = 0.295, *p* = 0.010).

**Table 1:** Demographic information and clinical characteristics of the three groups.

Item	PD	PD-mild	PD-moderate	HC	p_(PD vs. NC)_	p_(PD-mild vs. NC)_	p_(PD-moderate vs. NC)_	p_(PD-mild vs. PD-moderate)_
Number (M/F)	75(42/33)	43(27/16)	32(15/17)	37(17/20)	0.316	0.131	0.938	0.170
Age (years)	58.95 ± 9.55	57.30 ± 8.58	61.16 ± 10.46	58.05 ± 8.78	0.634	0.717	0.166	0.076
Duration (month)	23.69 ± 20.74	18.00 ± 16.61	31.34 ± 23.39	−	−	−	−	0.005
Education (years)	7.03 ± 3.71	7.35 ± 3.92	6.59 ± 3.42	7.64 ± 3.46	0.406	0.726	0.238	0.375
HY stages	1.63 ± 0.60	1.15 ± 0.23	2.27 ± 0.25	−	−	−	−	0.000
UDPRS	25.12 ± 15.10	19.84 ± 11.90	32.22 ± 16.17	−	−	−	−	0.001
UDPRS-III	15.3 ± 10.42	11.12 ± 7.09	20.94 ± 11.58	−	−	−	−	0.000
MMSE	26.08 ± 3.73	26.35 ± 2.98	26.53 ± 2.64	25.38 ± 5.07	0.748	0.846	0.703	0.996
HAMD-17	5.82 ± 6.11	6.30 ± 6.30	8.78 ± 6.90	2.70 ± 3.13	0.000	0.002	0.000	0.060

Data are shown as means ± SD. PD, Parkinson’s disease; PD-mild, Parkinson’s disease at 1.0–1.5 stage; PD-moderate, Parkinson’s disease at 2.0–2.5 stage; HC, Healthy controls; M, male; F, female; HY, Hoehn and Yahr; UPDRS, Unified Parkinson’s Disease Rating Scale; UDPRS-III, the motor part of UPDRS; MMSE, Mini-Mental State Examination; HAMD-17, 17-item Hamilton Depression Scale; −, Data not available.

### Group Differences of Regional Homogeneity

**Figure 1:**
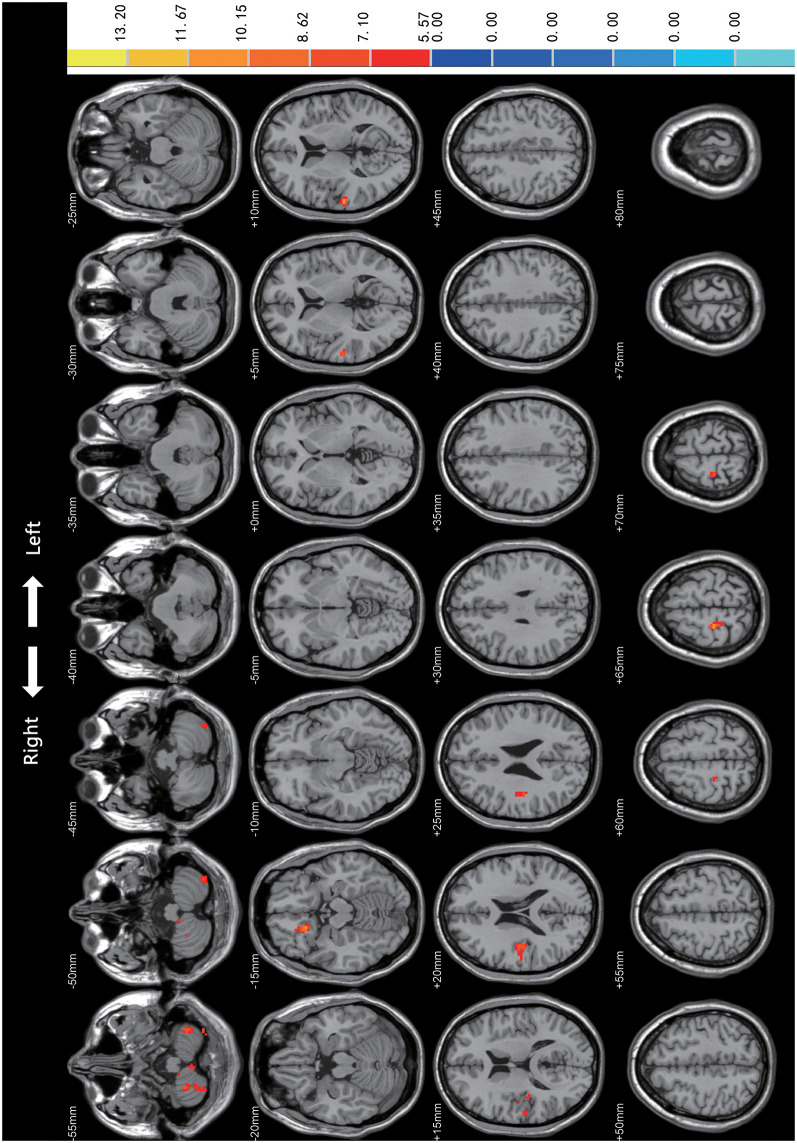
Comparison of Regional Homogeneity (ReHo) among PD-mild, PD-moderate, and HC groups. Significant differences were revealed in the following brain regions: bilateral cerebellum (Cerebellum_8/9_R, Cerebellum_8_L, Cerebellum_Crus2_L), right superior orbital gyrus, right rectus, right superior temporal gyrus, right Rolandic operculum, right postcentral gyrus, and the right precentral gyrus (*p* < 0.05).

Statistical analyses were observed using an automated anatomical atlas (AAL) template^2^. ANCOVA revealed the significant differences between the PD-mild, PD-moderate, and HC groups in the following brain regions: the bilateral cerebellum (Cerebellum_8/9_R, Cerebellum_8_L, Cerebellum_Crus2_L), the right superior orbital gyrus (Frontal_Sup_Orb_R), the right rectus (Rectus_R), the right superior temporal gyrus (Temporal_Sup_R), the right Rolandic operculum (Rolandic_Oper_R), the right postcentral gyrus (Postcentral_R), and the right precentral gyrus (Precentral_R; Figure 1).

**Figure 2:**
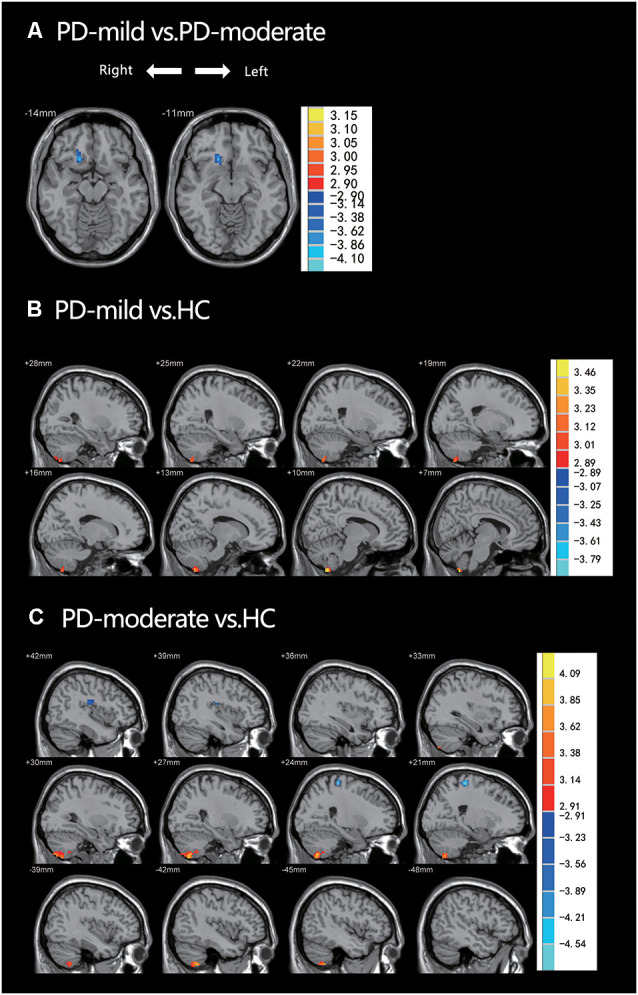
**(A)** PD-mild vs. PD-moderate groups; ReHo had decreased in the Frontal_Sup_Orb_R and the Rectus_R. **(B)** PD-mild vs. HC; ReHo had increased in the Cerebellum_8_R. **(C)** PD-moderate vs. HC; ReHo had increased in the bilateral cerebellum and decreased in the Temporal_Sup_R, Rolandic_Oper_R, Superior Temporal Gyrus, Postcentral_R, and Precentral_R. Table 2 shows more specific information relating to the significant brain regions.

In the *post hoc* analysis (Table 2 and Figure 2), only one cluster survived when comparing the PD-mild group to the PD-moderate group, with the cluster extending from the Frontal_Sup_Orb_R to the Rectus_R (Figure 2A). When compared to the HC group, the PD-mild group presented with increased ReHo values in the Cerebellum_8_R (Figure 2B). The PD-moderate group presented with increased ReHo values in the bilateral cerebellum (Cerebellum_8_R, Cerebellum_8_L), and reduced ReHo values in the Temporal_Sup_R, Rolandic_Oper_R, Superior Temporal Gyrus, Postcentral_R, and Precentral_R (Figure 2C). The results were corrected by the AlphaSim program with a voxel-level *p* < 0.005, cluster-level *p* < 0.05 for a two-tail test and cluster size >24 voxels.

**Table 2:** Brain regions showing significant ReHo differences between paired groups from the PD-mild, PD-moderate, and HC groupings.

Groups	Brain region (AAL template)	Cluster size	Peak MNI coordinates (x y z)	*t*-value
PD-mild < PD-moderate	Frontal_Sup_Orb_R Rectus_R	24	18 21 −12	−3.8828
PD-mild > HC	Cerebellum_8_R	57	9 −63 −63	3.5775
PD-moderate > HC	Cerebellum_8_R Cerebellum_9_R	143	9 −51 −63	4.3226
	Cerebellum_8_L	24	−42 −51 −57	3.8637
PD-moderate < HC	Temporal_Sup_R Rolandic_Oper_R	56	60 −24 9	−4.3039
	Precentral_R Postcentral_R	30	21 −30 66	−4.8632

*L, left hemisphere; R, right hemisphere; AAL, automated anatomical atlas; MNI, Montreal neurological institute; sup, superior; t-value, t statistic of *post hoc* analysis in two sample t-test*.

### Correlative Analysis

**Figure 3:**
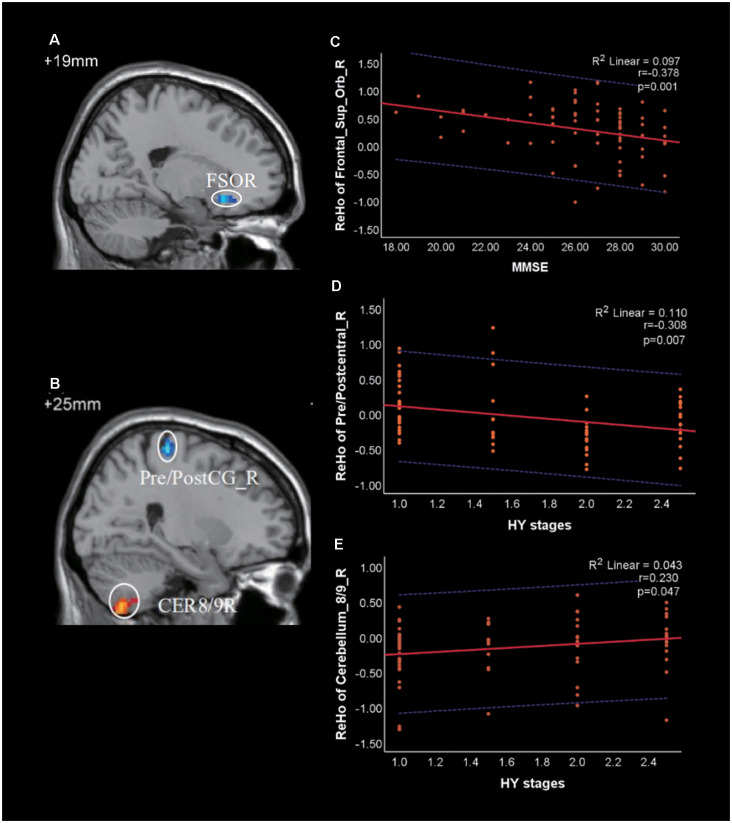
Correlation of Regional Homogeneity (ReHo) between brain regions and clinical scale scores in patients with PD. **(A)** FSOR, Frontal_Sup_Orb_R; **(B)** CER8/9R, Cerebellum_8/9_R; Pre/PostC_R, Pre/postcentral_R. **(C)** The ReHo value of Frontal_Sup_Orb_R was negatively correlated with Mini-Mental State Exam (MMSE). **(D,E)** The ReHo value of Pre/Postcentral_R and Cerebellum_8/9_R were negatively and positively correlated with HY stages, respectively. The red solid line shows the existence of a significant correlation, while the blue dotted line depicts the 95% prediction interval for the red solid line.

Using the two PD groups, we calculated Spearman correlation coefficients between the ReHo values of the clusters showing significant differences and clinical scale scores, including disease duration, UPDRS, UPDRS-III, HY, MMSE, and HAMD-17 scores. The brain regions related to the above clinical data have been marked in Figures 3A and 3B. Negative correlations were identified between the following pairs: ReHo values of the Frontal_Sup_Orb_R and MMSE scores (Figure 3C, *r* = −0.378, *p* = 0.001), ReHo values of the Pre/Postcentral_R and HY stages (Figure 3D, *r* = −0.308, *p* = 0.007). The results were corrected by Bonferroni’s correction (0.05/6).

Correlations were also identified between the following pairs: ReHo values of the Cerebellum_8/9_R and HY stages (Figure 3E, *r* = 0.230, *p* = 0.047), ReHo values of the Pre/Postcentral_R and UPDRS-III (*r* = −0.252, *p* = 0.029). However, both of them were not significant under the Bonferroni’s correction.

## Discussion

In this study, we used the ReHo value as an indicator to investigate differences in local synchronization among HC, PD-mild, and PD-moderate groups. We also analyzed correlations between different brain regions and clinical scale scores. We attempted to investigate the imaging and functional features of PD within HY 2.5 stages in order to assist with the early diagnosis and treatment of PD patients.

In comparison with the PD-moderate group, we observed reduced ReHo values in the PD-mild group in the Frontal_Sup_Orb_R extending to the Rectus_R. Previous structural and functional MRI studies have revealed that frontal regions, such as the insula, orbitofrontal, olfactory sulcus, and dorsolateral frontal, are more apt to change than posterior regions in the early stage of PD (Yang et al., 2013; Claassen et al., 2016; Chaudhary et al., 2020). The Frontal_Sup_Orb, located in the ventral surface of the prefrontal lobe, is part of the orbitofrontal cortex (OFC); the Rectus is sometimes incorporated into the OFC. It has become clear that the OFC is related to the production of emotions, sensory integration, and hedonic experiences. These are complex neural mechanisms in which information flows from the OFC and other brain regions, especially the anterior cingulate cortex (ACC) and the amygdala (Kringelbach, 2005; Deng et al., 2016). When the OFC malfunctions, subjects may experience a number of mental or behavioral disorders, such as cognition dysfunction, emotion disorder, a failure to make decisions, social dysfunction, and impulse-control disorders (Damasio et al., 1994; Rudebeck and Rich, 2018). For patients with PD, these disorders are frequently associated with non-motor symptoms and tend to occur during the early stages of the disease (Pfeiffer et al., 2014; Bhattacharjee, 2018). In the present study, we observed differences in the ReHo values in the Frontal_Sup_Orb and the Rectus between the PD-mild and PD-moderate groups; correlation analysis suggested that this brain region was associated with cognition (Figure 3C). Collectively, our data indicate that changes in the Frontal_Sup_Orb and the Rectus may serve as a differential indicator for mild and moderate PD.

Compared with the HC group, the PD-mild and PD-moderate group showed increased ReHo values in cerebellum_8/9_R and cerebellum_8_L region. Over recent years, the role of the cerebellum in PD has received increasing amounts of research attention. A number of pathological, morphological, and functional, studies have revealed that the cerebellum plays an important role in the pathological and compensatory effects of PD with regards to both motor and non-motor symptoms (Wu and Hallett, 2013; Stöger et al., 2017; Li M. et al., 2019; Miterko et al., 2019). Deep brain stimulation (DBS) of the basal ganglia or the pedunculopontine nucleus may work well on PD patients if there is a connection to the cerebellum, thus indicating the compensatory role of the cerebellum in PD, at least indirectly (Miterko et al., 2019). In addition, it is now widely accepted that the cerebellum plays a role in perceptual and connective processing (Baumann et al., 2015; Adamaszek et al., 2017; Kansal et al., 2017). The posterior cerebellar lobes, particularly lobules VI and VII, are known to be involved in a range of cognitive tasks, including memory and execution (Stoodley et al., 2012; Li M.-G. et al., 2019). Collectively, these lines of evidence indicate that the cerebellum may contribute to both motor and non-motor symptoms in PD patients. In our study, the increased ReHo values observed in the cerebellum may form part of the compensatory mechanism in PD.

When compared with the HC group, patients in the PD-moderate group showed a more extensive increased ReHo value in the cerebellum than the PD-mild group; furthermore, this increase was noted in both the right and left cerebellum. Correlation analysis indicated that the ReHo value in the Pre/Postcentral_R decreased as disease deteriorated, while the ReHo value in the cerebellum increased (not significant under the strict Bonferroni’s correction). Consistent with previous findings, our study suggested that the increase in ReHo value in the cerebellum forms part of a compensatory effect for abnormalities in the cerebral cortex. We believe that larger increases in ReHo value in the cerebellum of the PD-moderate group referred to a wider form of compensation. It appears that the compensation for cortical changes moved from right to the left in the cerebellum; however, whether this direction was inherent or related to the left- and right-onset of PD, remains unclear and requires further investigation.

In comparison with the HC group, subjects in the PD-moderate group exhibited reduced ReHo values in the cerebral cortex while subjects in the PD-mild group did not, including the Rolandic_Oper_R, Temporal_Sup_R, Postcentral_R, and Precentral_R regions. Some previous studies have reported structural or functional alterations in the Rolandic Operculum in PD patients (New et al., 2015; Xu et al., 2018; Liu et al., 2019; Wang T. et al., 2020). One previous study focused on the voice network of PD patients with vocalization impairment; this work identified alterations in the Rolandic Operculum (New et al., 2015). In the current study, we observed reduced ReHo values in the PD-moderate group when compared to the HC group, thus providing further support to the growing number of studies that have revealed the importance of the Rolandic Operculum in PD. Lesions or gray matter atrophy in the Rolandic Operculum have been related to movement disorders or tonic contractions of the perioral muscle; these changes can induce swallowing dysfunction or dysarthria (Tonkonogy and Goodglass, 1981; Biesbroek et al., 2016; Shen et al., 2016; Wang Y. et al., 2020). In addition, an fMRI study concluded that the Rolandic Operculum was involved in speech production and motor control (Behroozmand et al., 2015). Swallowing dysfunction has been frequently observed in PD patients and is evident in up to 100% of patients with advanced stages (Simons, 2017). However, this form of dysfunction is not just observed in the late stages of PD; mild oropharyngeal symptoms and esophageal dysfunction are quite common events in the early stages of PD (Potulska et al., 2003; Simons, 2017). Dysphagia or speech disturbances are frequently observed in patients with different stages of PD. The most common speech impairment is hypokinetic dysarthria, a disorder that is characterized by articulatory deficits and phonetic monotony (Jankovic, 2008; Ricciardi et al., 2016; Melchionda et al., 2020). Combined with previous findings, our current analyses indicate that the reduced ReHo values in the Rolandic Operculum of patients in the PD-moderate group were most likely related to the swallowing and speech disorders observed in PD patients. The neocortex has been shown to be involved in Braak stages 5 and 6 of PD; this relates to the progressive deposition of Lewy bodies in the brain (Kon et al., 2020). Previous functional and *in vivo* metabolic studies have also suggested that abnormal cortical activity can be observed in the early stages of PD (Brooks, 2010; Choe et al., 2013). Combined with these earlier findings, our data suggest that cases of early PD in HY stages 2.0–2.5 may already have reached Braak stages 5 and 6 in terms of pathology.

There were some limitations to the present study that need to be considered. Firstly, we compared different HY stages of PD using a cross-sectional study instead of a longitudinal study. Secondly, although we identified a functional change in the Rolandic Operculum in patients in the PD-moderate group, we were unable to perform further correlation analysis due to the lack of clinical assessment data relating to swallowing function or speech disorders. Thirdly, we did not include PD patients with HY stages 3.0–5.0; this was because of the small number of patients in these stages and due to the risk of dopamine against withdrawal syndrome (Rabinak and Nirenberg, 2010) in these patients.

## Conclusion

In conclusion, our current findings suggest that the HC, PD-mild, and PD-moderate, groups exhibited different ReHo alterations in the bilateral cerebellum, right superior orbital gyrus, right rectus, right superior temporal gyrus, right Rolandic operculum, right postcentral gyrus, and right precentral gyrus. The superior orbital gyrus and rectus may serve as differential indicators for mild and moderate PD. Patients with moderate PD had greater scope for ReHo alterations in the cortex and compensation in the cerebellum than those with mild PD. PD patients in HY stages 2.0–2.5 may already be at Braak stages 5 and 6 in terms of pathology. Our findings revealed differences in the resting-state brain functional pattern in PD patients at different HY stages and may help us to elucidate the neural function and the early diagnosis of PD.

## Data Availability Statement

The raw data supporting the conclusions of this article will be made available by the authors, without undue reservation.

## Ethics Statement

The studies involving human participants were reviewed and approved by Ethics Committee of the 2nd Xiangya Hospital, Central South University. The patients/participants provided their written informed consent to participate in this study.

## Author Contributions

JL, YZ, LZ, MW, ZM, CS, and FZ: data collection. JL, HL, QS, and SC: data analysis. JL, HL, and TW: manuscript writing. CT: project development and manuscript revision. All authors contributed to the article and approved the submitted version.

## Conflict of Interest

The authors declare that the research was conducted in the absence of any commercial or financial relationships that could be construed as a potential conflict of interest.

## Footnotes

^1^http://www.restfmri.net

^2^http://www.gin.cnrs.fr/tools/aal
